# Elucidation of the composition, antioxidant, and antimicrobial properties of essential oil and extract from *Citrus aurantifolia* (Christm.) Swingle peel

**DOI:** 10.1016/j.sjbs.2024.103987

**Published:** 2024-04-05

**Authors:** Nandang Permadi, Mohamad Nurzaman, Febri Doni, Euis Julaeha

**Affiliations:** a Doctorate Program in Biotechnology, Graduate School, Universitas Padjadjaran, Bandung 40132, Indonesia; b Department of Biology, Faculty of Mathematics and Natural Sciences, Universitas Padjadjaran, Jatinangor 45363, Indonesia; c Department of Chemistry, Faculty of Mathematics and Natural Sciences, Universitas Padjadjaran, Jatinangor 45363, Indonesia

**Keywords:** *Citrus aurantifolia*, Essential oil, Antimicrobial, *Musa* spp., Micropropagation, Antioxidant

## Abstract

The most effective methodologies for generating *Musa* spp. explants involve the utilization of plant tissue culture micropropagation techniques. However, the pervasive challenge of microbial contamination significantly impedes the successful micropropagation of *Musa* spp. This study examined the antioxidant and antibacterial characteristics of the essential oil (LPO) and extract (LPE) obtained from the peel of *Citrus aurantifolia*. Additionally, we explored their mechanisms against common microbial contaminants in *Musa* spp. micropropagation. Using gas chromatography-mass spectrometry, we identified 28 components in LPO, with δ-limonene, β-pinene, citral, trans-citral, β-bisabolene, geranyl acetate, and α-pinene as the primary constituents. Meanwhile, liquid chromatography-mass spectrometry detected 17 components in LPE, highlighting nobiletin, tangeretin, scoparone, sinensetin, tetramethylscutellarein, 5-demethylnobiletin, and pyropheophorbide A as the predominant compounds. Evaluation using the DPPH and ABTS methods revealed the IC_50_ values for LPE at 0.66 ± 0.009 and 0.92 ± 0.012 mg/mL, respectively, indicating higher antioxidant activity compared to LPO, with IC_50_ values of 3.03 ± 0.019 and 4.27 ± 0.023 mg/mL using the same methods. Both LPO and LPE exhibited antimicrobial activities against all tested contaminant microorganisms through in vitro assays. Mechanistic investigations employing time-kill analysis, assessment of cell membrane integrity, and scanning electron microscopy (SEM) revealed changes in the morphological characteristics of the tested microbial contaminants, intensifying with increased concentration and exposure duration of LPO and LPE. These alterations led to substantial damage, including cell wall lysis, leakage of intracellular components, and subsequent cell death. Consequently, LPO and LPE emerge as promising alternatives for addressing microbial contamination in banana tissue cultures.

## 1 Introduction

Plantains and bananas (*Musa* spp.) hold substantial importance as food commodities in developing countries (Hamill and Rames, 2018, Israeli and Lahav, 2016, Soorianathasundaram et al., 2015). At present, the most effective methods for producing *Musa* spp. explants involve the utilization of plant tissue culture micropropagation method (Nathan and Scobell, 2012, Nurzaman et al., 2022, Wirakarnain et al., 2008). This technique has the capability to produce explants that are free from diseases, possess superior-quality banana explants, and yield high-quality planting material (George et al., 2008, Misra and Saema, 2016). Additionally, it expedites the creation of a large quantity of consistent plants in a brief period, fostering strong plant development in the following growth cycle (Agbadje et al., 2021, Ferdous et al., 2015). However, contamination presents considerable hurdles when growing *Musa* spp. using tissue culture technique (Cobrado and Fernandez, 2016, El-Banna et al., 2021, Msogoya et al., 2012, Permadi et al., 2023).

Contamination can occur due to the presence of microorganisms in the phyllosphere, rhizosphere, and endosphere parts of the plants (Ganen et al., 2010, Rawal and Keharia, 2019). The primary bacterial contaminants responsible for the contamination of *Musa* spp. tissue culture include *Bacillus subtilis*, *Klebsiella pneumoniae*, *Paenibacillus* spp., and *Pseudomonas* spp. (El-Banna et al., 2021, Ganen et al., 2010, Hamill and Rames, 2018, Thomas et al., 2008). Meanwhile, fungal contaminants that often contribute to contamination include *Aspergillus flavus*, *A. niger*, *Fusarium* spp., and *Trichoderma* spp. (Agbadje et al., 2021, Animasaun et al., 2022, Kithaku et al., 2019, Mokbel et al., 2017). Inadequate treatment of explant contamination can result in less regeneration capacity, decreased callus formation, and suppressed adventitious shoot growth (Cobrado and Fernandez, 2016, El-Banna et al., 2021). Moreover, the existence of microbiological impurities further contributes to elevated plant mortality rates, variations in growth (such as decreased shoot proliferation and roots), tissue necrosis, and even the death of plant explants (Permadi et al., 2023).

*Citrus aurantifolia* (Christm.) Swingle, a herbaceous plant widely cultivated in Indonesia, is valued for its medicinal properties (Jain et al., 2020, Julaeha et al., 2022a). The lime peel is rich in a diverse spectrum of phytochemical compounds, encompassing polyphenols, flavonoids, saponins, steroids, and terpenoids, among others (Aripin et al., 2015, Julaeha et al., 2022b, Panwar et al., 2021). In particular, the lime peel essential oil (LPO) and extract (LPE) exhibited such bioactivities as antioxidant, antimicrobial, antiviral, anticancer, antidiabetic, anti-inflammatory, and insecticide (Indriyani et al., 2023, Julaeha et al., 2023, Permadi et al., 2021). However, there is a lack of detailed information on how LPO and LPE act to inhibit the proliferation of microorganisms that commonly contaminate *Musa* spp. micropropagation.

This study seeks to uncover the compositions of LPO and LPE, assess their antioxidant and antimicrobial properties, and investigate how they combat bacterial and fungal strains responsible for contamination in *Musa* spp. micropropagation. Furthermore, it employs techniques such as time-kill analysis, assessment of cell membrane integrity, and scanning electron microscopy (SEM) to elucidate the mechanistic actions of LPO and LPE derived from *C. aurantifolia* peel in managing contamination during *Musa* spp. micropropagation.

## 2 Materials and methods

### 2.1 Materials

**Fig. 1:**
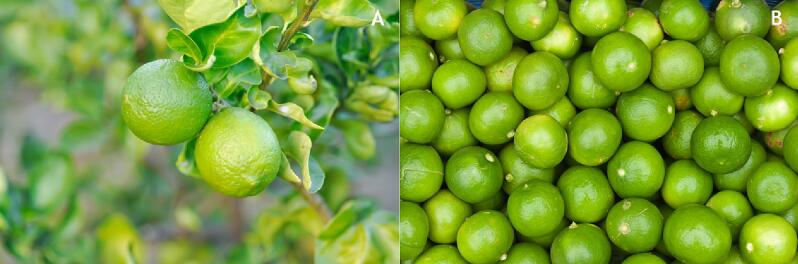
*C. aurantifolia* (Christm.) Swingle. (**A**): A close look at lime fruits in plants; (**B**): Lime fruits collected for this experiment (Photos courtesy of Nandang Permadi).

The limes were obtained from plantations located in the Banyuwangi Regency of East Java, Indonesia (Fig. 1). The microbes used in this study were obtained from the Microbial Cultures Collection at the Central Laboratory, Universitas Padjadjaran. The strains employed include *B. subtilis* ATCC 6633 and *Paenibacillus* sp., both Gram-positive bacteria, as well as *K. pneumoniae* ATCC 2357 and *Pseudomonas* sp., both Gram-negative bacteria. Additionally, four fungal strains were included: *A. flavus*, *A. niger*, *Fusarium* sp., and *T. viride*.

### 2.2 LPO and LPE extraction

The LPO was acquired through Stahl's hydrodistillation technique, adhering to the procedures outlined by Julaeha et al. (2023). The lime peel underwent distillation at 100 °C for 4 h. The resulting LPO was then dehydrated using anhydrous sodium sulfate and preserved in a tightly sealed vial in the refrigerator. Meanwhile, in order to acquire LPE, the maceration method was employed following the process used by Nurzaman et al. (2022). The lime peel was cleansed, desiccated at ambient temperature, and pulverized into a fine powder with a size 150 μm. The powder underwent complete immersion in an ethanol solvent, followed by an overnight soak. Afterward, the extract was collected. The residue underwent successive immersions in ethanol, with this process being repeated for up to three consecutive periods of 24 h each. Concentration of the extract was achieved using a Buchi R-200 rotary evaporator maintained at 50 °C.

### 2.3 Chemical characterization of LPO and LPE

The LPO examination was carried out using a gas chromatography-mass spectrometry (GC–MS/MS) setup, comprising an Agilent GC Type 7890A coupled with an MS Type 5975C. The DB-35 MS GC Column, measuring 30 m in length, 0.25 mm in inner diameter, and coated with a 0.25 μm film thickness, was employed for the analysis. Employing GC–MS/MS in the electron impact ionization mode with an energy of 70 eV within the *m*/*z* range of 50 to 500 u, the injector and ion source temperatures were set at 250 and 280 °C, respectively. The temperature protocol for the oven involved initiating at 50 °C, maintaining it for 1 min, raising it to 130 °C at a rate of 5 °C per minute for 0.5 min, and subsequently ramping up to 250 °C at a rate of 15 °C per minute, where it remained for 10 min. Helium served as the carrier gas, consistently flowing at a rate of 1.0 mL/min. Identification of LPO components was carried out using retention indices (RI) via the NIST 2.0 library version.

The LPE samples underwent analysis through ultra-performance liquid chromatography (UPLC) using specific equipment: the LC: ACQUITY UPLC® H-Class System by Waters, USA, along with a Xevo G2-S QTof mass spectrometer, also from Waters, USA, to generate high-resolution data. The C18 column (1.8 μm 2.1x100 mm, ACQUITY UPLC® HSS, Waters, USA) was employed, maintained at temperatures of 50 °C (column) and 25 °C (room). For the liquid chromatography (LC) analysis, the mobile phase comprised water with 5 mM ammonium formic acid and acetonitrile with 0.05 % formic acid. A step gradient was utilized at a flow rate of 0.2 mL/min over 23 min. Initially, a 5 μL LPE injection was filtered through a 0.2 μm syringe filter. Mass spectrometry (MS) analysis involved electrospray ionization (ESI) in positive mode, covering a mass range of 50–1200 *m*/*z*. The source and desolvation temperatures were set at 100 and 350 °C, respectively. Gas flow rates for cone and desolvation were 0 L/hr and 793 L/hr, while collision energy ranged from 4 to 60 eV. Masslynx software version 4.1 was employed for data collection, analysis, and instrument control.

### 2.4 Evaluation of antioxidant activities of LPO and LPE

The antioxidant capabilities of LPO and LPE were assessed through the 2,2-diphenyl-2-picrylhydrazyl (DPPH) radical scavenging assay outlined by Wang and Jiang, 2018, Wang et al., 2018 and the 2,2′-azinobis(3-ethylbenzothiazoline-6-sulfonic acid (ABTS) radical scavenging technique as detailed by Haj Ammar et al. (2012), with some modifications.

During the DPPH radical scavenging assay, 0.1 mL of both LPO and LPE samples, at different concentrations, were mixed with 2 mL of DPPH (0.21 mM in 95 % ethanol) in duplicate. Following this, the blend was incubated in darkness for 60 min, and the absorbance at 517 nm was measured post-incubation. Ethanol served as the control instead of LPO and LPE, while ascorbic acid acted as the standard antioxidant chemical. The DPPH free radical scavenging activity was determined using the subsequent equation:

$$\%DPPH\;scavenging\;activity=\frac{A_{\mathrm{control}}-A_{\mathrm{sample}}}{A_{\mathrm{control}}}x100\%$$

Whereas *A_sample_* represents sample absorbance, *A_control_* stands for control absorbance. The antioxidant activity was quantified by determining the IC_50_, which represents the concentration (expressed in mg/mL) of LPO and LPE necessary to block 50 % of the DPPH. The IC_50_ was determined by plotting the DPPH scavenging activity percentages against varying concentrations of LPO and LPE.

For the ABTS radical scavenging assay, a fresh 0.9 mL ABTS solution was combined with 0.1 mL of the sample (LPO and LPE) diluted in methanol. Following a 6-minute mixing period, absorbance at 734 nm was measured and noted as *A_sample_*. Moreover, a control trial was carried out in a similar manner using a solution devoid of the tested substance, yielding an absorbance value noted as *A_control_*. The ABTS free radical activity of each solution was then assessed by computing the percentage of inhibition through the subsequent formula:

$$\%ABTS\;scavenging\;activity=\frac{A_{\mathrm{control}}-A_{\mathrm{sample}}}{A_{\mathrm{control}}}x100\%$$

The assessment of antioxidant potential was measured through IC_50_, representing the concentration of LPO and LPE (mg/mL) required to achieve a 50 % decrease in the initial ABTS concentration. Ascorbic acid served as the reference standard. The measurements were conducted in duplicate.

### 2.5 Evaluation of antimicrobial activity of LPO and LPE

#### 2.5.1 Antibacterial and antifungal activity of LPO and LPE

The antimicrobial effects of LPO and LPE were evaluated using the agar diffusion method described by Goñi et al. (2009), with modifications. To summarize, a 100 μL microbial suspension containing approximately 1 × 10^7^ CFU/mL was uniformly spread on MHA medium for bacterial assessment and PDA medium for fungal evaluation. The negative control involved 2 % DMSO, while amoxicillin and ketoconazole served as positive controls in similar trials. A paper disc of 6 mm diameter soaked with 20 μL of the sample was placed on the surface of the plates, followed by incubation at 37 °C for 24 h. Inhibited growth zones on each plate were identified and their diameters measured in mm.

#### 2.5.2 Determination of MIC, MBC, and MFC

The MIC was determined following Da Silveira et al. (2014) method with minor modifications. The sample was diluted in a 5 % (v/v) DMSO solution. Serial dilutions were prepared in sterile NB, ranging from 0.020 to 50 mg/mL, with a two-fold concentration decrease at each step. Then, a 180 μL solution was mixed with a 20 μL microbial suspension (approximately 10^7^ CFU/mL) in 96-well plates and incubated at 37 °C for 24 h. The MBC and MFC were determined by transferring 50 μL from wells showing no visible microbial growth onto NA plates, followed by another 24-h incubation at 37 °C.

### 2.6 Demonstration of antimicrobial mechanisms of LPO and LPE

#### 2.6.1 Time-kill analysis

Time-kill analysis, as explained by Joray et al. (2011), was used to look into how well LPO and LPE killed bacteria and fungi. After incubating the microorganisms in NB medium at 37 °C for 8 h, they were centrifuged and reconstituted in sterile saline to reach a concentration of around 10^5^ CFU/mL. The microbial mix was then combined with NB medium containing 5 % DMSO and varying concentrations of LPO/LPE: control, MIC, and 2 × MIC. These inoculants were agitated and cultivated at 37 °C. Samples were withdrawn at set intervals, diluted in sterile saline, and cultured on MHA/PDA medium. After incubating for 24 h at 37 °C, the CFU count was determined through enumeration.

#### 2.6.2 The integrity of cell membrane

The method reported by Du et al. (2012) was used to release cell components into the supernatant, with some changes. Microorganisms derived from a 100 mL culture medium underwent separation via centrifugation at 3500 × g for 10 min, followed by triple washing and suspension in a 0.1 M PBS solution at pH 7.4. These cells were introduced into a 100 mL NB media solution containing 5 % DMSO and LPO/LPE at three varying concentrations: control, MIC, and 2 × MIC. The microbial suspension was maintained in a stirred incubator at 37 °C for durations of 0, 1, 2, 3, 4, 5, 6, and 7 h. Post-treatment, cells were subjected to centrifugation at 3500 × g, and the supernatant was scrutinized at 260 nm using a UV/Vis Spectrophotometer (UH-5300). The suspension, after a two-minute exposure to the strains under investigation, was absorbed using the same PBS solution with identical oil concentrations. Cells left untreated were adjusted with PBS for comparative analysis.

#### 2.6.3 Morphological changes analysis

The SEM technique was employed to examine the morphological alterations, following the methodology outlined by Bajpai et al. (2013), with some minor adjustments. Microorganisms were grown in NB medium at 37 °C for 8 h, then spun at 5000 revolutions per minute. The resulting cells were suspended in a phosphate buffer solution (PBS) at around 10^8^ CFU/mL and pH 7.4. This suspension was mixed with varying concentrations of LPO and LPE, including control, MIC, and 2 × MIC. DMSO constituted 5 % (v/v) of the total volume, followed by incubation at 37 °C for 4 h. Centrifugation at 8000 × g for 5 min and two washes with 0.1 M PBS ensued. The cells were later immersed in a solution of 2.5 % (v/v) glutaraldehyde in 0.1 M PBS, sputter-coated with gold using an ion coater for 2 min, and observed using a scanning electron microscope (JSM IT-200).

### 2.7 Statistical analysis

The collected data underwent analysis of variance (ANOVA) using IBM SPSS Statistics 23 software. Upon detecting a significant effect (p < 0.05), subsequent testing was conducted employing Duncan's multiple range test (DMRT) to ascertain variations among treatments.

## 3 Results

### 3.1 LPO and LPE extraction

In this study, 1.96 kg of *C. aurantifolia* peel was used each time for hydrodistillation to produce 9.46 g LPO, resulting in a yield of 0.48 ± 0.014 %. Meanwhile, the preparation of LPE was carried out using the maceration method for 3 x 24 h. A total of 686.75 g *C. aurantifolia* peel simplicia was used to produce 112.92 g LPE, resulting in a yield of 16.45 ± 0.064 %.

### 3.2 Chemical characterization of LPO and LPE

**Fig. 2:**
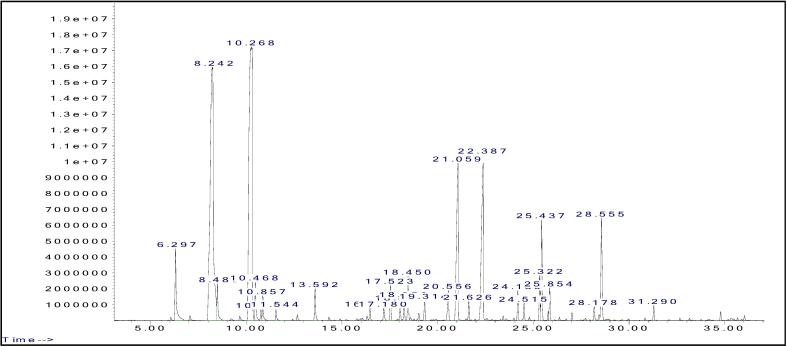
GC–MS/MS chromatogram of the LPO.

**Fig. 3:**
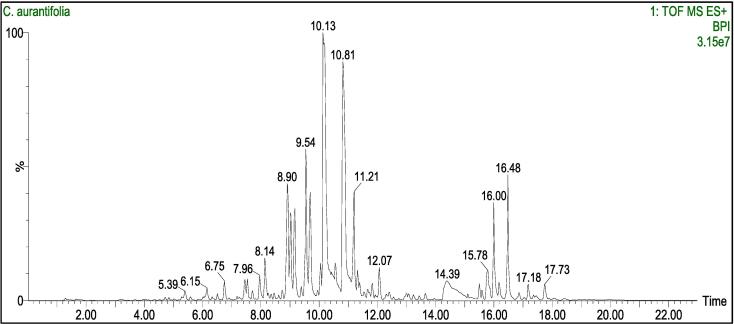
LC-MS/MS chromatogram of LPE.

The primary components of LPO were determined using GC–MS/MS analysis, showcasing their chromatogram in Fig. 2. Similarly, the main components of LPE were identified via LC-MS/MS analysis, illustrating their chromatogram in Fig. 3.

In Table 1, GC–MS/MS analysis identified 28 compounds, with seven predominant constituents in LPO being δ-limonene (29.69 %), β-pinene (26.31 %), citral (9.94 %), *trans*-citral (7.98 %), β-bisabolene (3.72 %), geranyl acetate (3.22 %), and α-pinene (2.91 %). Meanwhile, Table 2 displays 17 compounds detected by LC-MS/MS, seven highlighting nobiletin (23.42 %), tangeretin (17.15 %), scoparone (13.29 %), sinensetin (10.66 %), tetramethylscutellarein (9.46 %), 5-demethylnobiletin (5.64 %), and pyropheophorbide A (5.58 %) as the primary components of LPE.

**Table 1:** Components of the lime peel oil.

**No.**	**Compounds**	**RT (min)**	**Area (%)**	**SI (%)**
1	**α-Pinene**	6.297	2.91	96
2	**β-Pinene**	8.242	26.31	97
3	β-Myrcene	8.488	1.22	64
4	**δ-Limonene**	10.268	29.69	99
5	β-Phellandrene	10.468	1.25	91
6	o-Cymene	10.754	0.31	95
7	β-Ocimene	10.857	0.73	97
8	γ-Terpinene	11.544	0.40	96
9	Linalool	13.592	1.04	97
10	Citronellal	16.459	0.39	94
11	Norcarane	17.180	0.42	38
12	Terpinen-4-ol	17.523	1.11	95
13	Pulegone	18.038	0.56	46
14	Decanal	18.250	0.63	91
15	α-Terpineol	18.450	1.41	86
16	Geranial	19.314	0.63	96
17	Geraniol	20.556	1.17	97
18	***trans*-Citral**	21.059	7.98	91
19	δ-Elemene	21.626	0.51	97
20	**Citral**	22.387	9.94	96
21	Elemene	24.189	0.83	91
22	Geranyl propionate	24.515	0.53	91
23	α-Bergamotene	25.322	1.24	97
24	**Geranyl acetate**	25.437	3.22	74
25	γ-Elemene	25.854	0.95	98
26	Germacrene D	28.178	0.46	96
27	**β-Bisabolene**	28.555	3.72	86
28	α-Farnesene	31.290	0.43	91
**Total**			99,72	

RT, retention time; SI, silimarity index.

**Table 2:** Components of the lime peel extract.

**No.**	**Compounds**	**RT (min)**	**Area (%)**	**Neutral Mass (Da)**
1	Furoin	5.39	0.72	192.042252
2	Aurantio-obtusin β-D-glucoside	6.15	0.66	492.126770
3	Coumarin	6.75	0.64	146.036774
4	Citropten	7.96	0.80	206.057907
5	Rubidate	8.14	1.78	304.094696
6	**Scoparone**	8.90	13.29	206.057907
7	**Sinensetin**	9.54	10.66	372.120911
8	**Tetramethylscutellarein**	9.70	9.49	342.110352
9	**Nobiletin**	10.13	23.42	402.131470
10	**Tangeretin**	10.81	17.15	372.120911
11	**5-Demethylnobiletin**	11.21	5.64	388.115814
12	Scopoletin	12.07	1.11	192.042252
13	Tributyl citrate acetate	14.39	3.94	402.225372
14	Pheophorbide A	16.00	3.75	592.268555
15	**Pyropheophorbide A**	16.48	5.58	534.263062
16	Nimbolinin D	17.18	0.65	620.298523
17	Erucamide	17.73	0.73	337.334473

RT, retention time.

### 3.3 Antioxidant activities of LPO and LPE

**Fig. 4:**
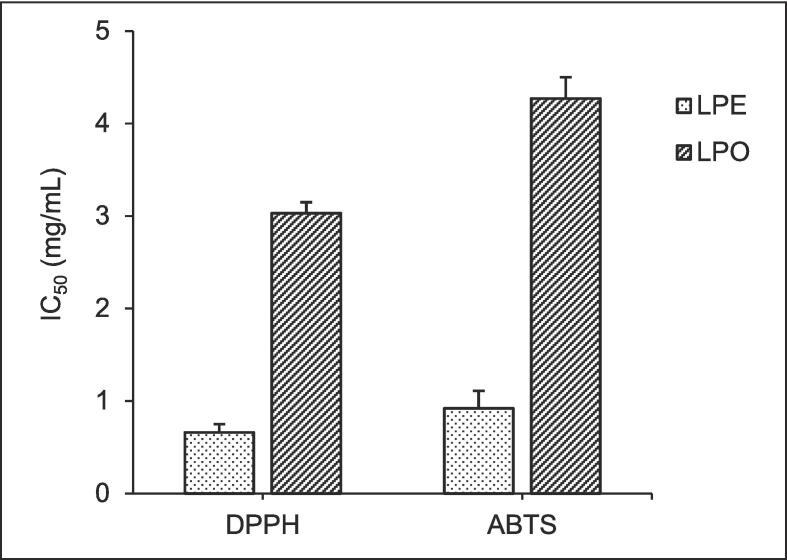
Antioxidant activity of LPO and LPE.

The antioxidant potentials of LPO and LPE were evaluated using DPPH and ABTS radical-scavenging assays. Fig. 4 demonstrates how the capacity of LPO and LPE to neutralize 50 % of free radicals quantifies their antioxidant effects. The IC_50_ values for LPE assessed through the DPPH and ABTS methods were 0.66 ± 0.009 and 0.92 ± 0.012 mg/mL, respectively. In contrast, the IC_50_ values for LPO using the same methods were 3.03 ± 0.019 and 4.27 ± 0.023 mg/mL, respectively. LPE has higher antioxidant activity compared to LPO in both the DPPH and ABTS methods.

### 3.4 Antibacterial activities of LPO and LPE

Various amounts of LPO and LPE were examined for their effects on four bacterial species responsible for contamination in *Musa* spp. plant tissue culture. Limonene, the primary constituent of LPO, is employed as a reference for comparison. Table 3 displays the inhibition zones diameter (IZD) for LPO, LPE, and limonene in comparison to amoxicillin against the tested bacterial contaminants. The IZD values exhibited a positive correlation with the concentration, indicating a direct relationship. It was found that LPO, LPE, and limonene worked better on Gram-positive bacteria (*B. subtilis* and *Paenibacillus* sp.) than on Gram-negative bacteria (*K. pneumoniae* and *Pseudomonas* sp.). The antibacterial efficacy against all examined bacteria differed among the different LPO and LPE. The IZD ranging from 36.9 to 48.1 mm at a 100 % concentration of LPO demonstrated that it had superior antibacterial activity to other samples.

**Table 3:** Antibacterial activities of LPO and LPE expressed as inhibition zones diameters (mm).

**Samples**	**Concentrations (%)**	**Gram-positive**		**Gram-negative**	
		*Bacillus* *subtilis*	*Paenibacillus* sp.	*Klebsiella pneumoniae*	*Pseudomonas* sp.
Lime peel oil	100	48.1 ^g^	44.9 *^m^*	43.3^i^	36.9^j^
	75	36.0^f^	34.0 ^l^	32.6 ^h^	27.6^i^
	50	28.4^e^	25.9^j^	21.6 ^g^	18.4^f^
	25	13.5^b^	12.3^d^	10.8 ^cd^	9.2^c^
Lime peel extract	100	26.5^d^	18.8 ^g^	15.8^e^	17.7^f^
	75	19.9^c^	14.2^e^	11.7^d^	13.3^e^
	50	13.5^b^	8.9^c^	7.6^b^	8.2^c^
	25	7.1^a^	3.0^a^	4.0^a^	2.5^a^
Limonene	100	26.4^d^	31.9 ^k^	21.1 ^g^	23.1 ^h^
	75	19.5^c^	24.0^i^	15.9^e^	17.4^f^
	50	13.1^b^	16.0^f^	10.5^c^	11.3^d^
	25	7.1^a^	7.6^b^	7.0^b^	5.7^b^
Positive control (Amoxicillin)	25	26.3^d^	21.5 ^h^	18.7^f^	20.2 ^g^
Negative control (DMSO)	2	*N.a*	*N.a*	*N.a*	*N.a*
SEM		3.255	3.293	3.043	2.621
P-values		<0.050	<0.050	<0.050	<0.050

Means with same letters within a column do not differ significantly according to DMRT (p < 0.050). SEM, standard error of means.

### 3.5 Antifungal activities of LPO and LPE

Four types of fungi, known to infect *Musa* spp. plant tissue cultures, were utilized to test LPO and LPE at various concentrations. Limonene, the primary component of LPO, served as the comparative reference. Table 4 illustrated the diameter of inhibition zones (IZD) for LPO, LPE, and limonene against these fungi in comparison to ketoconazole. The IZD values exhibited a concentration-dependent increase. Our results revealed varied antifungal effects among the different concentrations of LPO and LPE against all tested fungal contaminants. Notably, LPO was more effective at killing fungi than other samples, creating inhibition zones that were between 20.0 and 44.6 mm wide at 100 % concentration.

**Table 4:** Antifungal activities of LPO and LPE expressed as inhibition zones diameters (mm).

**Samples**	**Concentrations (%)**	***Aspergillus flavus***	***Aspergillus niger***	***Fusarium* sp.**	***Trichoderma viride***
Lime peel oil	100	35.4 ^k^	20.0 ^g^	36.8^i^	44.6^j^
	75	25.4^i^	14.9^f^	28.2 ^h^	31.8 ^h^
	50	16.7 ^g^	9.2^c^	17.9^f^	21.6^f^
	25	7.7^c^	4.6^b^	9.1^c^	11.1^d^
Lime peel extract	100	10.1^d^	9.7 ^cd^	10.8^d^	11.9^d^
	75	5.6^b^	4.5^b^	5.6^b^	7.8^c^
	50	1.5^a^	1.4^a^	1.5^a^	5.6^b^
	25	*N.a*	*N.a*	*N.a*	1.1^a^
Limonene	100	29.1^j^	25.5 ^h^	27.4 ^h^	35.1^i^
	75	21.9 ^h^	19.5 ^g^	21.4 ^g^	26.3 ^g^
	50	14.6^f^	13.1^e^	13.8^e^	17.0^e^
	25	5.6^b^	5.2^b^	6.0^b^	6.7^bc^
Positive control (Ketoconazole)	25	11.9^e^	10.6^d^	11.0^d^	11.2^d^
Negative control (DMSO)	2	*N.a*	*N.a*	*N.a*	*N.a*
SEM		3.032	2.121	3.104	3.650
P-values		<0.050	<0.050	<0.050	<0.050

Means with same letters within a column do not differ significantly according to DMRT (p < 0.050). SEM, standard error of means.

### 3.6 MIC, MBC, and MFC of LPO and LPE

LPO and LPE act as microbial inhibitors, bactericides, and fungicides against four tested bacterial species and four tested fungal species, as seen in Table 5. The LPO has MICs ranging from 0.20 to 3.13 mg/mL and MBC/MFCs ranging from 0.39 to 12.5 mg/mL. In contrast, LPE shows MICs ranging from 0.98 to 7.81 mg/mL and MBC/MFCs ranging from 3.91 to 31.5 mg/mL. Limonene, the primary compound in LPO, exhibits similar MICs and MBC/MFCs to LPO, ranging from 0.20 to 6.25 mg/mL and 0.39 to 12.5 mg/mL, respectively. Notably, LPO demonstrates heightened fungicidal activity against *T. viride*, displaying the lowest MIC and MBC values. Conversely, LPE shows the most potent bactericidal activity against *B. subtilis*, showcasing the minimum MIC and MBC values. Consequently, the ensuing section focuses on uncovering the antimicrobial mechanism by administering LPE treatment to *B. subtilis* and LPO to *T. viride*.

**Table 5:** MIC, MBC, and MFC of LPO and LPE against tested bacteria and fungi.

**Microorganisms**	**MIC (mg/mL)**			**MBC/MFC (mg/mL)**		
	**Lime peel oil**	**Lime peel extract**	**Limonene**	**Lime peel oil**	**Lime peel extract**	**Limonene**
**Bacteria**						
*Bacillus subtilis*	0.39^a^	0.98^a^	1.56^ba^	0.78^a^	3.91^a^	1.56^ba^
*Klebsiella pneumoniae*	3.13^b^	7.81^b^	6.25^c^	12.50^b^	31.25^c^	12.50^c^
*Paenibacillus* sp.	1.56^ab^	1.95^a^	0.78^a^	12.50^b^	15.63^b^	6.25^b^
*Pseudomonas* sp.	1.56^ab^	3.91^a^	3.13^b^	12.50^b^	15.63^b^	12.50^c^
**Fungi**						
*Aspergillus flavus*	0.78^a^	1.95^a^	0.20^a^	1.56^a^	15.63^b^	0.78^a^
*Aspergillus niger*	0.39^a^	7.81^b^	0.78^a^	0.78^a^	31.25^c^	0.78^a^
*Fusarium* sp.	0.39^a^	1.95^a^	0.20^a^	0.78^a^	31.25^c^	0.39^a^
*Trichoderma viride*	0.20^a^	1.95^a^	0.39^a^	0.39^a^	15.63^b^	1.56^a^
SEM	0.35	0.98	0.74	2.13	3.57	1.86
P-values	<0.050	<0.050	<0.050	<0.050	<0.050	<0.050

Means with same letters within a column do not differ significantly according to DMRT (p < 0.050). SEM, standard error of means.

### 3.7 Effect of LPO and LPE on the viability of microbial contaminants

**Fig. 5:**
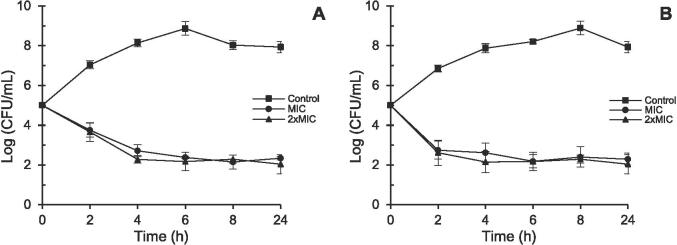
Time-kill analysis of *B. subtilis* and *T. viride*. (**A**): *B. subtilis*; (**B**): *T. viride*.

Time-kill assays were used to figure out how quickly LPO and LPE were inactivated. The results are shown in Fig. 5 as logarithms of the number of viable cells. Fig. 5A illustrates *B. subtilis* subjected to LPE at MIC and 2 × MIC levels, featuring untreated *B. subtilis* as the control. Similarly, Fig. 5B displays *T. viride* exposed to LPO at MIC and 2 × MIC concentrations, with untreated *T. viride* serving as the control.

The *B. subtilis* population without treatment grew from 5.2 to 8.9 × Log_10_ CFU/mL, reaching a plateau after 6 h. Upon treatment, there was a rapid decline within the initial 4 h, stabilizing around 2.3 × Log_10_ CFU/mL thereafter. When treated at 2 × MIC, *B. subtilis* exhibited a growth curve resembling that of MIC treatment. In the case of untreated *T. viride*, the population increased from 5.1 to 9.0 × Log_10_ CFU/mL within 8 h, remaining relatively stable before gradually decreasing to 7.9 × Log_10_ CFU/mL after 24 h. Treated *T. viride* showed a significant decrease compared to the untreated sample. The number of viable *T. viride* cells treated at both MIC and 2 × MIC dropped to approximately 2.4 × Log_10_ CFU/mL within the initial two h and maintained this level afterward.

### 3.8 The effect of LPO and LPE on the cell membrane integrity

**Fig. 6:**
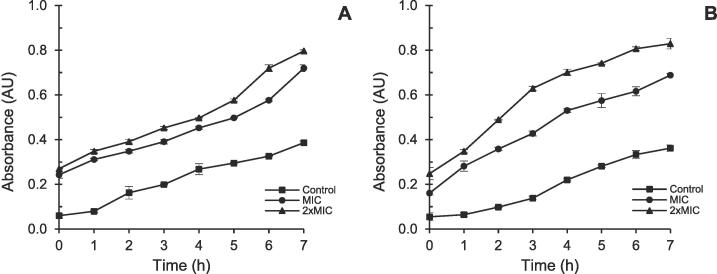
Effects of LPO and LPE on the cell membrane integrity of *B. subtilis* and *T. viride*. (**A**): *B. subtilis*; (**B**): *T. viride*.

The impact of LPO and LPE on the cell membrane integrity over a 7-h period is presented in Fig. 6. In Fig. 6A, B*. subtilis* was subjected to LPE at MIC and 2 × MIC concentrations, with untreated *B. subtilis* serving as the control. Similarly, Fig. 6B illustrates *T. viride* exposed to LPO at MIC and 2 × MIC concentrations, with untreated *T. viride* as the control.

The absorbance values for *B. subtilis* significantly increased, rising from 0.243 ± 0.016 to 0.720 ± 0.015 at MIC and from 0.270 ± 0.001 to 0.797 ± 0.006 at 2 × MIC. Meanwhile, the control showed an increase from 0.060 ± 0.011 to 0.386 ± 0.008. Additionally, there was a notable rise in the release of cellular components with increasing concentrations of LPE over time. Concerning *T. viride*, absorbance values ranged between 0.055 ± 0.008 and 0.362 ± 0.014 for the control, 0.161 ± 0.009 and 0.689 ± 0.008 for MIC, and 0.248 ± 0.027 and 0.829 ± 0.023 for 2 × MIC during the treatment period with LPO.

### 3.9 The effect of LPO and LPE on morphological changes

**Fig. 7:**
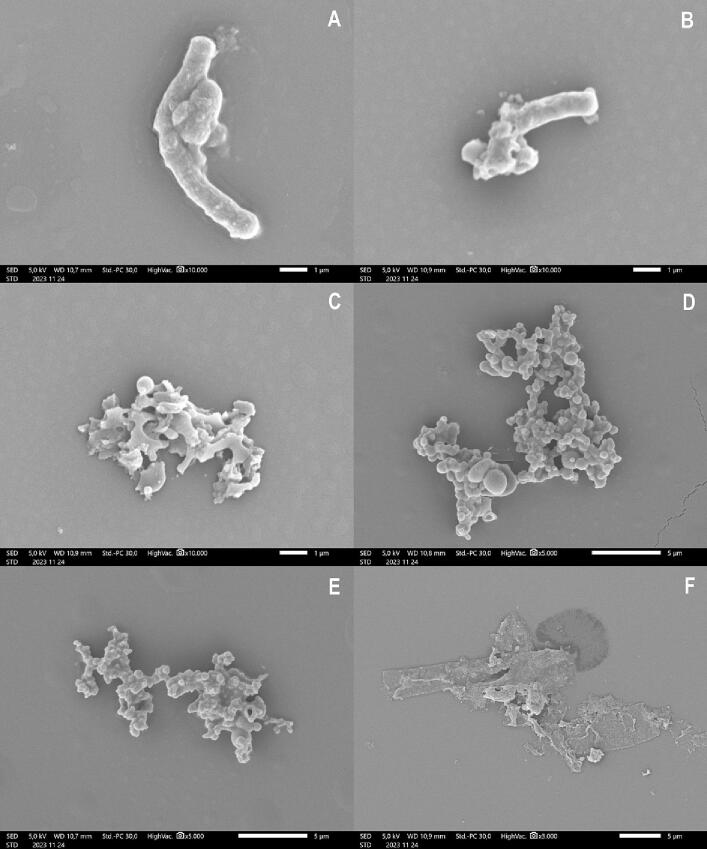
The effects of LPE on morphological changes of *B. subtilis*. (**A**): Untreated *B. subtilis*; (**B**): *B. subtilis* treated with LPE at MIC; (**C**): *B. subtilis* treated with LPE at 2 × MIC; and LPO on morphological changes of *T. viride* (**D**): Untreated *T. viride*; (**E**): *T. viride* treated with LPO at MIC; (**F**): *T. viride* treated with LPO at 2 × MIC.

*B. subtilis* was subjected to LPE at MIC and 2 × MIC concentrations, with untreated *B. subtilis* serving as the control. Similarly, *T. viride* underwent treatment with LPO at MIC and 2 × MIC concentrations, while untreated *T. viride* served as the control. SEM was utilized to examine the morphological alterations in both *B. subtilis* and *T. viride*, both treated and untreated, with the findings presented in Fig. 7.

Fig. 7A illustrates untreated *B. subtilis*, showcasing clear attributes such as consistent rod-shaped formations, undamaged surfaces, and lined cell walls. In contrast, Fig. 7B and 7C illustrate that most treated bacteria exhibited varying degrees of irregularity and shrinkage. Furthermore, at a 2 × MIC level, the treated bacteria showed more severe morphological damage on their cell walls compared to those treated at the MIC concentration. Fig. 7D depicts untreated *T. viride*, showcasing conidia with intact, smooth surfaces and irregular pyramidal shapes. Conversely, Fig. 7E and 7F display that most treated fungi exhibited irregularities and shrinkage to different extents. Additionally, at the 2 × MIC concentration, the treated fungi displayed more pronounced morphological destruction in their conidia compared to those treated at the MIC concentration.

## 4 Discussion

In this study, LPO isolation utilized the hydrodistillation method, yielding 0.48 ± 0.014 %. Comparable methods were employed by previous researchers in isolating LPO, yielding 0.21–2.00 % (Julaeha et al., 2023, Pratiwi et al., 2022, Sharma and Vashist, 2015). Furthermore, Varkey et al. (2013) used hydrodistillation in five different places and obtained essential oils from *C. aurantifolia* leaves and bark that were between 0.10 and 0.14 % and 0.30 % and 0.60 %, respectively. Meanwhile, LPE was acquired through the maceration method utilizing ethanol as the solvent, yielding 16.45 ± 0.064 % (Hegazy and Ibrahium, 2012, Nurzaman et al., 2022). This aligns with prior research that explored the extraction of flavonoid and polyphenolic compounds from *Citrus* peel using various organic solvents, demonstrating ethanol yields ranging from 8.27 % to 28.32 %. Similarly, Safdar et al. (2016) investigated diverse techniques and solvents for extracting *C. reticulata* peel, discovering that ethanol extraction produced a yield of 18.46 %. The variations in essential oil and extract yields can be attributed to several factors, such as fruit variety, seasonal influences, geographical location, fruit maturity, and climatic conditions (Bora et al., 2020, Lin et al., 2019). These elements have a notable impact on both the makeup and quantity of the LPO and LPE.

The GC–MS/MS analysis showed that the major compounds of LPO are δ-limonene (29.69 %), β-pinene (26.31 %), citral (9.94 %), *trans-*citral (7.98 %), β-bisabolene (3.72 %), geranyl acetate (3.22 %), and α-pinene (2.91 %). In earlier research, Aripin et al. (2015) found that the chemical makeup of *C. aurantifolia*’s LPO included δ-limonene (38.9 %), β-pinene (26.7 %), α-terpineol (8.3 %), terpinene-4-ol (4.3 %) and citral (3.6 %). Moreover, Pandiangan et al. (2021) confirmed that it was made up of δ-limonene (30.34 %), β-pinene (28.32 %), citral (5.32 %), and β-bisabolene (4.44 %). Additional marker compounds from *Citrus* species, such as α-terpineol, terpinen-4-ol, β-myrcene, and γ-terpinene, were identified but in minor quantities. It is important to highlight that differences in the makeup of constituents are notably impacted by factors such as the plants’ geographical location, the specific part of the plant sampled, and the method used for isolation (Darjazi, 2014, Md Othman et al., 2016). Considering these influential variables, the variations observed in the results remain reasonable.

The LC-MS/MS test showed that the main chemical compounds in LPE are nobiletin (23.42 %), tangeretin (17.15 %), scoparone (13.29 %), sinensetin (10.66 %), tetramethylscutellarein (9.46 %), 5-demethylnobiletin (5.64 %), and pyropheophorbide A (5.58 %). Previous studies have established nobiletin, tangeretin, scoparone, sinensetin, tetramethylscutellarein, and 5-demethylnobiletin as major polymethoxyflavones found in citrus fruit peels, such as oranges, mandarins, limes, and lemons (Huang et al., 2020, Nakanishi et al., 2019, Rodov et al., 2019; Wang et al., 2018, Wang and Jiang, 2018). Additionally, pyropheophorbide A and pheophorbide A, derivatives of chlorophyllide, were detected in fresh lime, where they function as natural catabolites involved in the degradation of chlorophyll *a* induced by ethylene (Kaewsuksaeng et al., 2019, Yin et al., 2016). Highlighting the significant variations in chemical components is crucial because the composition of plant extracts is profoundly shaped by plant genetics and various stressors like water levels, light exposure, and pest presence (Silvestre et al., 2019).

Secondary metabolites from plants, like those found in *C. aurantifolia*, exhibit various biological activities, notably acting as antioxidants. In this investigation, the antioxidant potential of LPO and LPE was assessed using ABTS and DPPH tests. The findings revealed that LPE demonstrated notably higher antioxidant activity against ABTS and DPPH radicals compared to LPO. Further analysis via LC-MS/MS affirmed the presence of flavonoid compounds—such as nobiletin, tangeretin, and 5-demethylnobiletin—in LPE, known for their antioxidant properties, contributing to its enhanced antioxidant activity.

Nobiletin, tangeretin, and 5-demethylnobiletin, known for their strong antioxidant abilities, utilize a method to counteract free radicals like DPPH and ABTS. These substances contain specific structures that aid in providing hydrogen atoms or electrons, vital for neutralizing the lone electrons present in these radicals (Hegazy and Ibrahium, 2012, Wang et al., 2018, Wang and Jiang, 2018). When encountering the DPPH radical, these molecules participate in hydrogen atom transfer, intercepting the unpaired electron and forming a stabilized compound (Chen et al., 2020, Mitani et al., 2021). In the same way, nobiletin, tangeretin, and 5-demethylnobiletin donate electrons to the ABTS radical. This makes the radical less reactive by changing it into a form that is less reactive.

The antioxidative capabilities of nobiletin, tangeretin, and 5-demethylnobiletin are underpinned by the phenolic groups within their chemical structures (Li et al., 2014, Wang et al., 2018, Wang and Jiang, 2018). These groups serve as critical sites for hydrogen or electron donation, enabling these compounds to neutralize free radicals like DPPH and ABTS effectively (Singh et al., 2021). By engaging in hydrogen atom transfer or electron donation, these flavonoids disrupt the reactivity of radicals, rendering them inert or less harmful (Li et al., 2022). Their ability to scavenge free radicals underscores their potential for preventing cellular damage and highlights their significance as natural antioxidants with potential health benefits.

*C. aurantifolia* is widely reported to possess bioactivity, including antimicrobial properties. LPO and LPE both exhibit antibacterial and antifungal properties, although LPO has superior efficacy to LPE. This heightened activity is attributed to the diverse array of active chemical components within LPO, among which limonene plays a notable role. However, while limonene displays moderate antimicrobial activity on its own, studies by van Vuuren and Viljoen (2007) highlighted the potential for enhanced antimicrobial effects when limonene is present within essential oils, hinting at possible synergistic interactions that amplify its biological impact (Djabou et al., 2013, Lin et al., 2021). Such synergies among constituents in essential oils have been frequently documented.

LPE has the ability to impede bacterial growth through bioactive properties like flavonoids, terpenoids, and citrus-derived compounds (Latupeirissa et al., 2022, Thapa et al., 2022). These components exhibit antimicrobial traits, disrupting crucial bacterial cellular functions. The observed impact stems from their ability to interfere with the cell wall or compromise the integrity of the cell membrane (Mohammed et al., 2021). Additionally, these compounds can disrupt bacterial metabolism, including protein synthesis and DNA replication, which are essential for bacterial growth and development (Chanthaphon et al., 2008, Değirmenci and Erkurt, 2020). The ability of LPE to inhibit bacterial growth is often associated with the antioxidant properties of these compounds, which can induce oxidative stress within bacterial cells and disrupt their overall cellular functions (Indriyani et al., 2023, Mohammed and Ayoub, 2016). Additionally, LPO and LPE exhibited greater efficacy against Gram-positive bacteria compared to their Gram-negative counterparts. This variation might be attributed to differences in the structure of bacterial cell wall. Gram-negative bacteria have an outer membrane characterized by lipopolysaccharide molecules, creating a hydrophilic surface (Shakeri et al., 2014). The outer layer functions as a protective barrier, obstructing the entry of large molecules and hydrophobic substances into the targeted cell membrane. Consequently, this feature makes Gram-negative bacteria comparatively resilient against hydrophobic antibiotics (Kong et al., 2008).

On the contrary, LPO and LPE demonstrates notable antifungal properties. The discoveries underscore the swift and powerful influence of LPO, as it disrupts membrane integrity in *T. viride*, resulting in the release of nucleic acids and proteins through the membrane, eventually resulting in the demise of the cell. These outcomes align with SEM observations revealing substantial damage in *T. viride* following treatment with LPO. Moreover, when LPE was tested on *B. subtilis*, it showed a similar result. This efficacy likely results from LPO's and LPE’s ability to penetrate the cytoplasmic membrane, particularly targeting the mitochondrial membrane (Affes et al., 2022, Bakkali et al., 2008, Segaran et al., 2020). Subsequently, the affected mitochondria generate free radicals, triggering oxidation that damages lipids, proteins, and DNA (Karim et al., 2016, Liu et al., 2021).

## 5 Conclusion

Lime peel, once regarded as mere waste, has garnered significant attention due to its numerous bioactivities. In this study, essential oils and extracts were carefully obtained from *C. aurantifolia* to investigate their valuable properties. Notably, LPE showcased remarkable antioxidant activity surpassing that of LPO, while LPO exhibited superior antimicrobial efficacy compared to LPE. The outcomes underscored that the distinctive compositions of LPO and LPE are pivotal contributors to their respective antimicrobial and antioxidant properties. As a result, LPE and LPO emerge as promising alternatives for addressing contamination issues in crop micropropagation, including *Musa* spp. tissue culture.

## Funding

This work was funded by Kementerian Pendidikan, Kebudayaan, Riset, dan Teknologi Republik Indonesia, through Hibah Penelitian Disertasi Doktor (No. 3018/UN6.3.1/PT.00/2023), and Universitas Padjadjaran through Hibah ALG (No. 1549/UN6.3.1/PT.00/2023) awarded to Euis Julaeha.

## CRediT authorship contribution statement

**Nandang Permadi:** Data curation, Formal analysis, Investigation, Writing – original draft. **Mohamad Nurzaman:** Methodology, Project administration, Supervision, Writing – review & editing. **Febri Doni:** Methodology, Supervision, Validation, Writing – review & editing. **Euis Julaeha:** Conceptualization, Funding acquisition, Supervision, Validation, Writing – review & editing.

## Declaration of competing interest

The authors declare that they have no known competing financial interests or personal relationships that could have appeared to influence the work reported in this paper.
